# Immune Modeling Analysis Reveals Immunologic Signatures Associated With Improved Outcomes in High Grade Serous Ovarian Cancer

**DOI:** 10.3389/fonc.2021.622182

**Published:** 2021-03-05

**Authors:** Nicole E. James, Katherine Miller, Natalie LaFranzo, Erin Lips, Morgan Woodman, Joyce Ou, Jennifer R. Ribeiro

**Affiliations:** ^1^Program in Women's Oncology, Department of Obstetrics and Gynecology, Women and Infants Hospital of Rhode Island, Providence, RI, United States; ^2^Department of Obstetrics and Gynecology, Warren-Alpert Medical School of Brown University, Providence, RI, United States; ^3^Cofactor Genomics, San Francisco, CA, United States; ^4^Department of Pathology, Women and Infants Hospital of Rhode Island, Providence, RI, United States

**Keywords:** tumor immunology, immune profiling, LAG-3, ICOS, T regulatory cells, CTLA-4, PVRL2, ovarian cancer

## Abstract

Epithelial ovarian cancer (EOC) is the most lethal gynecologic malignancy worldwide, as patients are typically diagnosed at a late stage and eventually develop chemoresistant disease following front-line platinum-taxane based therapy. Only modest results have been achieved with PD-1 based immunotherapy in ovarian cancer patients, despite the fact that immunological responses are observed in EOC patients. Therefore, the goal of this present study was to identify novel immune response genes and cell subsets significantly associated with improved high grade serous ovarian cancer (HGSOC) patient prognosis. A transcriptomic-based immune modeling analysis was employed to determine levels of 8 immune cell subsets, 10 immune escape genes, and 22 co-inhibitory/co-stimulatory molecules in 26 HGSOC tumors. Multidimensional immune profiling analysis revealed CTLA-4, LAG-3, and T_regs_ as predictive for improved progression-free survival (PFS). Furthermore, the co-stimulatory receptor ICOS was also found to be significantly increased in patients with a longer PFS and positively correlated with levels of CTLA-4, PD-1, and infiltration of immune cell subsets. Both ICOS and LAG-3 were found to be significantly associated with improved overall survival in The Cancer Genome Atlas (TCGA) ovarian cancer cohort. Finally, PVRL2 was identified as the most highly expressed transcript in our analysis, with immunohistochemistry results confirming its overexpression in HGSOC samples compared to normal/benign. Results were corroborated by parallel analyses of TCGA data. Overall, this multidimensional immune modeling analysis uncovers important prognostic immune factors that improve our understanding of the unique immune microenvironment of ovarian cancer.

## Introduction

Epithelial Ovarian Cancer (EOC), is the most lethal of all gynecological malignancies, with approximately 21,750 women diagnosed, and 13,940 deaths from the disease in the United States in 2020 (1). The primary treatment regimen for advanced stage EOC consists of debulking surgery and platinum-taxane-based chemotherapy, with around 75% of patients achieving remission. However, a majority of patients experience recurrence within months to a few years, at which point the treatment response to traditional chemotherapy is low (2). To combat this chemoresistance, there has been a heavy focus on targeted therapies such as anti-angiogenic and poly (ADP-ribose) polymerase (PARP) inhibitors, which have shown promising results when used for maintenance or recurrent disease therapy. However, significant challenges still exist in producing long-term outcomes, with prognosis for advanced stage patients remaining poor (3–7).

Currently, several EOC clinical trials center upon immunotherapy (3). While melanoma and non-small cell lung cancer (NSCLC) patients have seen a great benefit from targeting immune checkpoint inhibitors such cytotoxic T lymphocyte-associated protein-4 (CTLA-4) or programmed cell death protein 1 (PD-1), EOC patients have only exhibited response rates of 10–15% (3). Despite these poor response rates, evidence suggests that EOC is an immunogenic cancer. It has been well-established that patients with a higher number of intratumoral T cells achieve a longer progression-free survival (PFS) and overall survival (OS) as compared to those with lower numbers (8). In addition, antigen-specific antibodies and tumor-reactive T cells have been detected in ovarian cancer (9). Thus, while evidence suggests that EOC patients could benefit from some form of immunotherapy, further research is needed in order to improve response rates.

While immune signatures may not be highly useful as predictive markers for immunotherapy in EOC, given the overall low response rates to currently studied PD-1 therapies, immune-related gene expression profiles may serve as prognostic indicators of response to frontline chemotherapy. Moreover, gaining an understanding of which immunological features in a tumor lead to improved outcomes could reveal opportunities for novel immunotherapeutic approaches. In ovarian cancer, studies have begun to explore the relationship between immune composition, immune-related genes, and clinical outcomes. Several studies have used computational analysis of tumors with publicly available gene expression data to report relationships between immune cell subsets or gene expression and prognosis (10–13). Yet other studies have employed immunohistochemical approaches to establish a relationship between intra-epithelial T cells and other immune cell subsets and survival (14, 15). However, a complete understanding of how the immune microenvironment responds to chemotherapy and how these changes relate to clinical outcomes is lacking.

In this present study, we performed a targeted transcriptomic analysis of immune cell content and coinhibitory and costimulatory receptor genes and their respective ligands in a cohort of 26 high grade serous ovarian cancer (HGSOC) tumors. This multidimensional analysis identified a novel combination of immune response genes and subsets that are associated with patient outcomes, and revealed unique correlations between specific immune genes in HGSOC. Specific genes were further analyzed using The Cancer Genome Atlas (TCGA) ovarian cancer cohort. These results highlight the potential utility of novel immune markers as prognostic factors or to inform future therapeutic strategies.

## Materials and Methods

### Ovarian Cancer Tissue

Stage IIIC, grade 3 serous ovarian cancer patients were selected based on duration of PFS (time from completing chemotherapy to first recurrence). One patient in the long PFS group had Stage IV disease that was only designated as Stage IV due to pelvic lymph node spread, which was easily resected. Eleven patients had a PFS ≥ 54 months (range 54–99+); eight patients had a PFS ≤ 9 months (range 0–9); and seven had an intermediate PFS (range 16–47). The median age of the patients in the long PFS group was 55 (range 42–91) and the median age was 67.5 (range 45–79) for the short PFS group (*p* = 0.1226). The median CA125 value for patients in the long PFS group was 155, and 1,808 for the short PFS group (*p* = 0.0346). Median HE4 levels were not calculated because only ten patients out of the 19 had known HE4 levels. Some patients were treated with maintenance bevacizumab, as noted (Table 1). Formalin-fixed, paraffin embedded (FFPE) tissue sections were prepared from each patient's residual tissue block from their primary debulking surgery. All tumors were naïve to chemotherapy. Before submission, the tissue was analyzed by a pathologist at Women & Infants Hospital to determine that they met minimum guidelines for cellularity (≥80%) and viability (≥20%). All tissue was obtained and data managed under The Women and Infants Hospital Institutional Review Board approval of protocol #1326537.

**Table 1:** Patient information.

**Group**	**Stage**	**Grade**	**Age range**	**Received maint. bev**	**PFS (with bev)**	**OS (with bev)**	**HE4**	**CA125**	**Debulking**
Long	IIIC	3	50–59	no	90+	90+	N/A	0–499	Optimal
Long	IIIC	3	50–59	no	93+	93+	0–499	0–499	Optimal
Long	IIIC	3	50–59	no	100+	100+	N/A	0–499	Optimal
Long	IIIC	3	40–49	no	84+	84+	0–499	0–499	Optimal
Long	IIIC	3	90–99	no	54	79+	100–499	1000–1499	Suboptimal
Long	IIIC	3	40–49	no	79+	79+	100–499	0–499	Optimal
Long	IIIC	3	70–79	yes	62 (51)	77 (66)+	N/A	0–499	Optimal
Long	IIIC	3	50–59	yes	76 (64)+	76 (64)+	0–499	500–999	Optimal
Long	IIIC	3	70–79	no	71+	71+	0–499	0–499	Optimal
Long	IV	3	50–59	no	71+	71+	0–499	0–499	Optimal
Long	IIIC	3	60–69	no	72+	72+	N/A	0–499	Optimal
Median			55		76 (72)	79 (79)		155	
Short	IIIC	3	60–69	no	2	4	N/A	0–499	Suboptimal
Short	IIIC	3	60–69	no	4	6	N/A	N/A	Optimal
Short	IIIC	3	70–79	no	6	14	N/A	0–499	Suboptimal
Short	IIIC	3	70–79	no	7	28	500–999	0–499	Optimal
Short	IIIC	3	60–69	yes	7 (2)	20 (15)	N/A	1500–1999	Suboptimal
Short	IIIC	3	40–49	yes	0 (0)	6 (0)	N/A	0–499	Suboptimal
Short	IIIC	3	60–69	no	9	18	0–499	3000+	Optimal
Short	IIIC	3	70–79	no	6	24	2000–2499	2500–2999	Optimal
Median			67.5		6 (5)	16 (14.5)		1808	
Intermediate	IIIC	3	70–79	no	16	38	N/A	2500–2999	Optimal
Intermediate	IIIC	3	60–69	yes	20 (9)	76 (67)+	N/A	1000–1499	Optimal
Intermediate	IIIC	3	50–59	yes	33 (22)	95 (84)	N/A	0–499	Optimal
Intermediate	IIIC	3	60–69	yes	47 (41)	51 (45)	0–499	0–499	Optimal
Intermediate	IIIC	3	60–69	no	17	47	N/A	0–499	Optimal
Intermediate	IIIC	3	80–89	no	24	29	1000–1499	0–499	Suboptimal
Intermediate	IIIC	3	60–69	no	31	56+	0–499	0–499	Optimal
Median			63		24 (22)	51 (47)		218	

*Clinical information for all 26 patients was determined, and median values calculated for each group (long, short, intermediate). In the case of patients who received maintenance bevacizumab, PFS and OS values from time of completion of bevacizumab are included in parentheses*.

### Cofactor ImmunoPrism® Assay

All stages of the assay were performed in a CAP-accredited, CLIA-licensed clinical laboratory. Schillebeeckx et al. (16) describes the machine learning approach used to generate “health expression models” for accurately identifying percentages of immune cell populations in tumor tissue. When developing the immune Health Expression Models used in the ImmunoPrism assay, all models were built using purified immune cell populations, and validated using flow cytometry. For example, the surface markers used to identify T_regs_ (CD4+/CD25+/CD127low/–/CCR4+) were based on those used by the Human Immunophenotyping Consortium (17). The CD4+/CD25+/CD127low/– subpopulation of cells has been shown to be significantly enriched for Foxp3 expression compared to the overall CD4+/CD25+ population, and additionally display higher suppressive capability (18). We therefore expect the CD4+/CD25+/CD127low/–/CCR4+ cells isolated to be significantly enriched for T_regs_. For macrophages, a serum-free *in vitro* differentiation model was used to generate M1 and M2 macrophages from peripheral blood monocytes. M1 macrophages were derived by activation with IFN-gamma and LPS, and M2 macrophages were derived by activation with IL-4, as described in The Biology of Cancer by Robert A. Weinberg (second edition) (19). Populations were confirmed by flow cytometry using the following markers: M1 macrophages are CD80+/CCR7+/CD206 low/CD209 low and M2 macrophages are CD206+/CD209+/CD80 low/CCR7 low.

LaFranzo et al. describes in detail the Cofactor Genomics predictive immune modeling workflow (16, 20), which is summarized as follows:

RNA Extraction: Unstained, unmounted FFPE sections from the same FFPE block were processed for RNA extraction using the Cofactor Prism Extraction Kit, following the manufacturer's suggested protocol.

Total RNA Quality Control: Unstained, unmounted FFPE sections from the same FFPE block were processed for RNA extraction using the Prism Extraction Kit (Cofactor Genomics, San Francisco, CA). Total RNA was evaluated for quality and quantity using the Bioanalyzer or TapeStation assay (Agilent, Santa Clara, CA), and the Qubit RNA HS or BR Assay (ThermoFisher, Waltham, MA). RNA concentration and quantity (in ng/μL and total ng) and quality (DV_200_, % of fragments above 200 nt) was evaluated to determine library input amount.

ImmunoPrism Library Preparation and Sequencing: Total RNA was processed for library construction by Cofactor Genomics according to the ImmunoPrism assay standard protocol for FFPE materials; 20–100 ng of RNA was used as input depending on sample quality. Libraries were sequenced as single-end 75 base pair reads on a NextSeq500 (Illumina, San Diego, CA) following the manufacturer's protocols.

ImmunoPrism Analysis: An individual ImmunoPrism report including expression characterization and immune cell quantification was provided for each sample processed. Samples were grouped according to clinical meta and outcomes data to generate ImmunoPrism Biomarker Reports, as appropriate.

Single analyte biomarkers were created using a linear threshold approach. All possible thresholds were considered and the threshold that jointly optimizes the leave-one-out specificity and sensitivity was chosen for each respective single analyte. This approach converges quickly to the population accuracy, even in small sample sizes, and is robust to class imbalance.

The multidimensional biomarker was created using machine learning to simultaneously consider multiple readouts from the ImmunoPrism assay. The process involves two steps: first, it chooses the highest performing combination of analytes, and then second, it chooses the highest performing machine learning hyperparameters to train the final model.

In the research-use-only version of the software, the top five analytes are considered, sorted by accuracy as an individual predictive readout and then by *p*-value. Next, all power set combinations of these five analytes were used to train Random Forest models with default hyperparameters. The analytes corresponding to the model with the lowest leave-one-out error were considered for the next step. In cases of ties, the model with more analytes was chosen. Using this final set of analytes, a grid search optimization over all hyperparameter values is performed. The model with the lowest leave-one-out error was used as the final model. The model's predictions of the left out samples were used to evaluate the performance of the final model.

### Immunohistochemistry

FFPE human ovarian tissue slides were baked at 2 h at 65°C. Slides were then washed in xylene, 100, 95, 70% ethanol, deoxygenated water, and FTA Hemagglutination Buffer. Antigen retrieval was performed using Antigen Retrieval Solution (1X) (Vector Laboratories, H-3300) and heated to 95°C for 20 min. Slides were then blocked with 5% horse serum in FTA Hemagglutination buffer and incubated overnight in PVRL2/Nectin-2 antibody (Cell Signaling, 95333S, 1:100) at 4°C. Anti-rabbit IgG Dylight 488 secondary antibody (Vector Laboratories, DI-488, 1:1000) was then applied to slides following incubation in the dark at room temperature for 1 h. Slides were washed between each step using FTA Hemagglutination buffer and cover-slipped with DAPI containing mounting medium (Vector Laboratories, H-1200).

### Image Analysis

Three randomly selected fields per case were selected based on DAPI staining and acquired with a spinning disk confocal Nikon Eclipse Ti microscope at 10x objective. Image analysis was performed on grayscale 8-bit images in ImageJ. Images were thresholded for specific staining and mean and maximum intensity was calculated. Representative images were taken using confocal microscopy at 20x objective.

### The Cancer Genome Atlas

The ovarian cancer dataset (TCGA-OV) with complete RNA-sequencing results (*n* = 378) from The Cancer Genome Atlas was obtained using GenomicDataCommons (version 1.12.0) and RStudio (R version 4.0.0) (21, 22). Fragments Per Kilobase of transcript per Million mapped reads (FPKM) values, vital status, and days to death/follow-up were obtained for correlation and survival analyses.

### Timer

TIMER2.0 (https://timer.cistrome.org) (23–25) was used to determine percentages of immune cells in TCGA-OV samples. CIBERSORT was used as the immune deconvolution method.

### cBioPortal

Mutation count data and RNA seq (V2 RSEM) data for TCGA-OV (Firehose Legacy) was downloaded from cBioPortal (https://cbioportal.org) (26, 27). Data was available for 185 samples.

### Statistics

The statistics reported for the ImmunoPrism assay were produced *via* leave-one-out cross validation. For a dataset limited in size, leave-one-out cross validation gives the best approximation to how an estimator will generalize to future, independent samples. The process works by iterating *n* times (where there are *n* datapoints), each time learning a threshold considering *n*-1 points and testing the prediction of the *n*th, left out, point. Then, all *n* predictions are considered to calculate prediction statistics.

Wilcoxon Rank Sum Test was used to determine differences in median values for age and CA125 levels. Cuzick trend test was used to evaluate differences in medians between “long”, “intermediate”, and “short” PFS groups. Kaplan-Meier curves were generated for TCGA survival data, and log-rank *p*-values along with hazard ratios and 95% confidence intervals were determined. Spearman rank correlation was used to determine correlations between genes and cell types. Two-tailed unpaired *t*-test was used to determine differences in gene expression levels between mutation count groups. Statistical analyses were performed in GraphPad Prism and R version 4.0.3. *p* < 0.05 was considered significant.

## Results

### Prognostic Biomarker Status in Epithelial Ovarian Cancer Tissue

**Figure 1:**
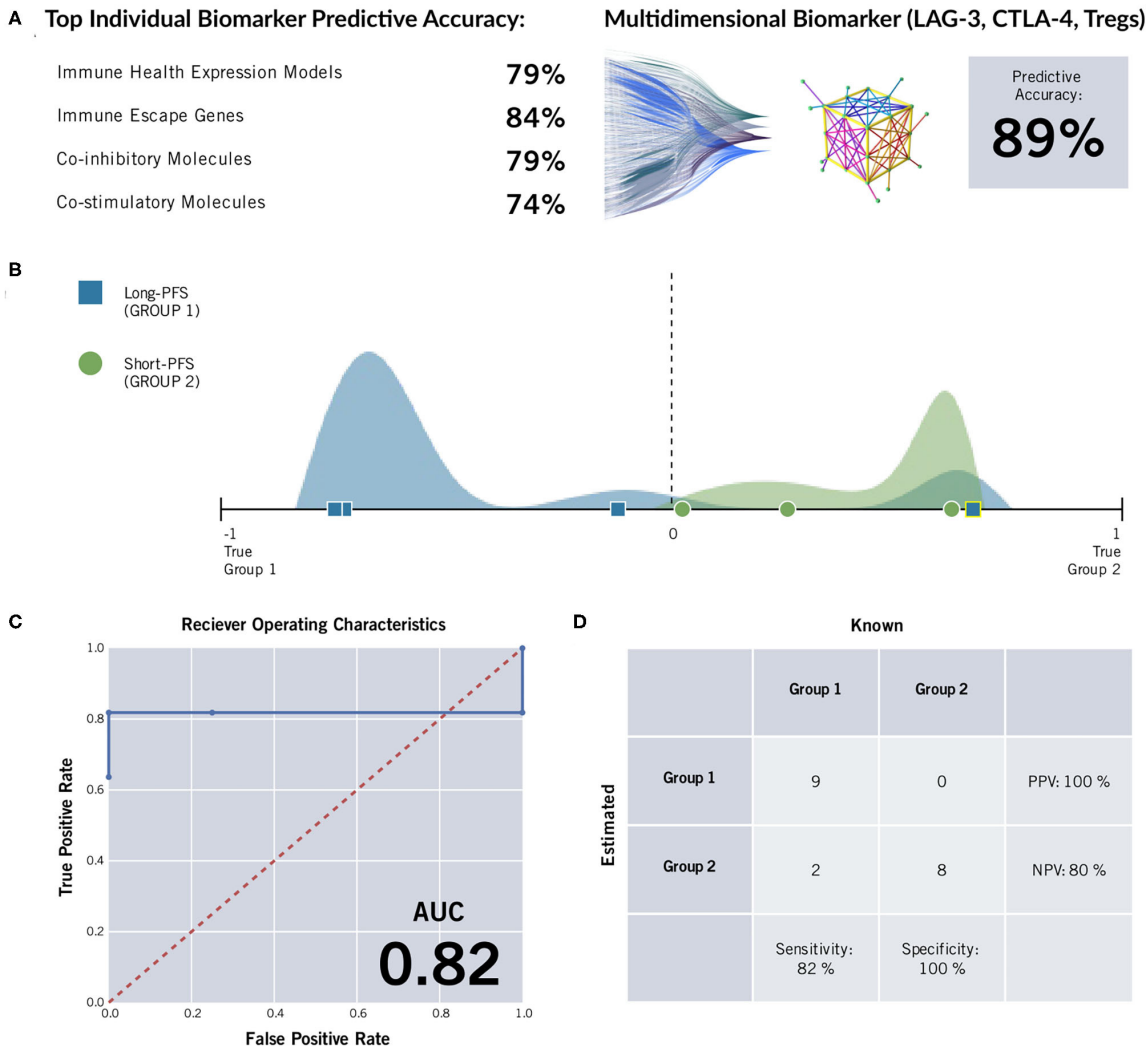
ImmunoPrism® Multidimensional Biomarker Assessment of high grade serous ovarian cancer. **(A)** The most predictive marker from immune cell types (“immune health expression models”) and each type of immune gene were less accurate than a multidimensional biomarker comprising LAG-3, CTLA-4, and T_regs_ at predicting length of progression free survival. **(B)** The distributions of individual samples from both cohorts were evaluated for future performance using the multidimensional biomarker as the classifier. Multidimensional values are plotted along the x-axis, and the frequency of sample values are visualized in the vertical space. The sample distribution visualization is approximate. The cohorts are distinguished by color (green and blue) and shape (circle and square), and estimations are distinguished by outline color (white: correct estimation, yellow: incorrect estimation.) The dashed line at value = 0 indicates the prediction threshold that separates the cohorts. Samples that were incorrectly estimated are highlighted in yellow, and listed in Supplementary Table 2. **(C)** A Receiver Operating Characteristic (ROC) Curve was generated for the multidimensional biomarker. For various thresholds of the biomarker, the True Positive Rate (y-axis) is plotted against the False Positive Rate (x-axis). The area under the curve (AUC) is included in the lower right corner. The random predictor is shown as a dashed red line. **(D)** A confusion matrix was generated for the multidimensional biomarker. This matrix was used to calculate positive predictive value (PPV), negative predictive value (NPV), specificity, and sensitivity of the multidimensional biomarker by comparing how the samples are classified based on the assay (rows) vs. their known label based on clinical data (28). The incorrectly estimated samples are the same as those noted and labeled in the Multidimensional Biomarker Assessment.

Twenty-six formalin fixed paraffin-embedded (FFPE) samples underwent ImmunoPrism immune profiling analysis (Cofactor Genomics, San Francisco, CA). Clinical data can be seen in Table 1. Nineteen samples that were stratified by PFS were included in the biomarker analysis to determine differences in a panel of immune genes and immune cell subtypes between the two groups (short vs. long PFS). Multidimensional immune profiling analysis revealed the combination of CTLA-4, LAG-3, and T_regs_ was significantly higher in patients with improved patient prognosis (Figure 1). Furthermore, individual analyte assessment revealed that LAG-3, CTLA-4, ICOS, and TNFRSF18 transcripts were significantly more abundant in the long PFS group compared to the short (*p* < 0.05). PD-1 also demonstrated a strong trend toward higher transcripts per million (TPM) in the long PFS group than in the short (*p* = 0.0575). The relationships between individual immune cell types and PFS did not reach the level of significance. Thresholds were determined for each cell type or gene, along with accuracy, positive predictive value (PPV), negative predictive value (NPV), sensitivity, and specificity (Table 2). RNA seq data is available through ArrayExpress (https://www.ebi.ac.uk/arrayexpress/) under accession number E-MTAB-9743.

**Table 2:** Analyte table displaying cell type percentages and TPM values for all genes analyzed in the ImmunoPrism assay.

**Analyte**	**Long PFS Median**	**Short PFS Median**	**Threshold**	***p*-value**	**Accuracy**	**PPV**	**NPV**	**Sensitivity**	**Specificity**
Multidimensional biomarker	−0.7	0.6	0	-	89	100	80	82	100
T_reg_ cells	5.3	3.2	4.1	0.0829	79	82	75	82	75
CD8+ T cells	2	0.8	1.2	0.1485	74	80	67	73	75
M2 macrophages	0.6	1	0.7	0.0829	63	56	70	62	64
CD4+ T cells	1.4	0.6	0.7	0.1864	63	70	56	64	62
M1 macrophages	0.1	0	0.1	0.2312	63	70	56	64	62
CD14+ monocytes	0.2	0.6	0.4	0.302	63	56	70	62	64
CD19+ B cells	5.9	4	5.3	0.8688	63	70	56	64	62
CD56+ NK cells	1.3	1.4	1.4	0.409	53	44	60	50	54
Total immune	17.6	14.9	-	-	-	-	-	-	-
CTLA4	3,191	1,912	2,343	0.039	84	83	86	91	75
PD-1	2,612	892	1,381	0.0575	74	80	67	73	75
ICOS	1,339	549	896	0.0475	63	70	56	64	62
PD-L1	1,648	900	1,388	0.0693	63	70	56	64	62
TIM-3	5,865	6,023	5,904	0.6797	58	50	64	50	64
OX40	4,580	3,727	4,010	0.0829	53	60	44	55	50
BTLA	1,385	767	938	0.3218	53	60	44	55	50
CD47	50,296	60,332	59,375	0.5089	53	44	60	50	55
ARG1	32	36	35	0.6797	53	44	60	50	55
IDO1	11,162	8,732	11,037	0.7412	47	56	40	45	50
LAG3	8,041	3,618	5,416	0.00 39	79	82	75	82	75
PDCD1LG2	2,852	1,500	1,999	0.0986	63	70	56	64	62
LGALS9	12,112	16,504	13,209	0.3218	63	56	70	62	64
CD96	6,364	2,863	4,419	0.3218	63	70	56	64	62
CD48	5,288	3,924	5,009	0.5089	63	70	56	64	62
CD244	355	412	360	0.8365	53	44	60	50	55
CD40LG	1,078	360	776	0.0693	74	80	67	73	75
CD28	1,483	914	1,011	0.1372	74	80	67	73	75
CD27	5,814	2,864	3,666	0.1864	74	80	67	73	75
TNFRSF18	4,811	2,402	3,168	0.039	68	73	62	73	62
CD70	918	593	875	0.1864	63	70	56	64	62
PVRL2	85,599	64,526	72,381	0.21 55	63	70	56	64	62
TNFRSF25	23,986	34,522	25,030	0.2155	63	56	70	62	64
TNFSF4	1,818	2,100	2,002	0.2477	63	56	70	62	64
TMIGD2	331	508	373	0.4828	63	56	70	62	64
CD80	2,318	1,994	2,218	0.6 203	63	70	56	64	62
CD40	22,051	14,557	19,788	0.7412	63	70	56	64	62
TNFSF18	75	87	81	1	63	56	70	62	64
HHLA2	92	265	106	0.3637	53	44	60	50	55
ICOSLG	421	381	419	0.6203	53	60	44	55	50
TNFSF15	307	436	410	0.9342	53	44	60	50	55
CD276	13,864	13,942	14,213	0.8688	47	40	56	50	45

*A “Multidimensional Biomarker” value was generated on the arbitrary scale of −1 to +1, where the “long PFS” group was placed on the negative axis. Accuracy, positive predictive value (PPV), negative predictive value (NPV), sensitivity, and specificity are displayed for each cell type or gene*.

### Relationship of Immune Genes and Cell Types With Progression-Free Survival

**Figure 2:**
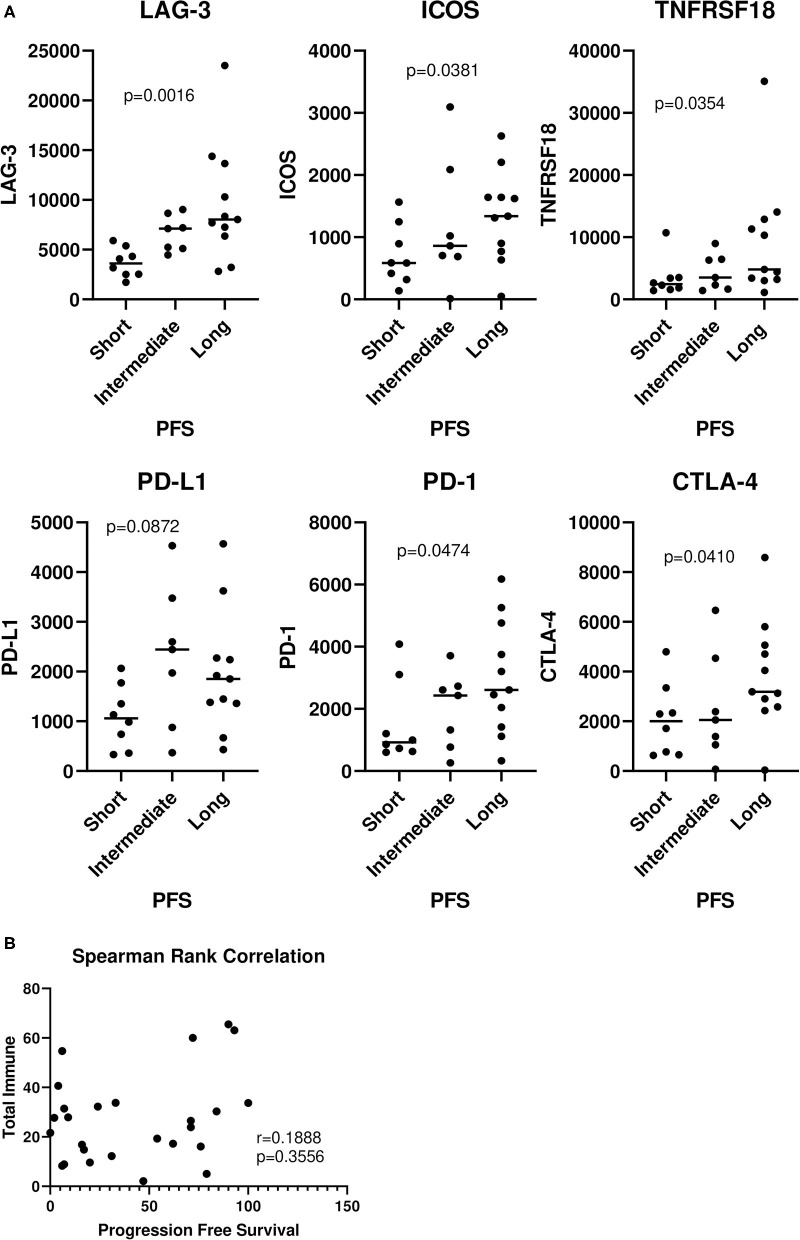
Relationship of immune genes and cell types with progression-free survival. The entire 26 patient cohort, including patients with short, intermediate, and long PFS were analyzed. **(A)** Values were plotted for each group (horizontal bar indicates median). Cuzick trend test was used to determine significant trends in the three groups. The horizontal variation in each group is a random jitter and not the actual PFS values. **(B)** “Total immune” percentages were correlated with PFS by Spearman rank correlation.

Next, we went on to analyze the entire cohort of samples, including those with short, intermediate, and long PFS. There were significant trends observed in the analysis of LAG-3 (*p* = 0.0016), ICOS (*p* = 0.0381), TNFRSF18 (*p* = 0.0354), PD-1 (*p* = 0.0474), and CTLA-4 (*p* = 0.0410) (Figure 2A). No significant trends were observed with any of the cell types and PFS (Supplementary Table 1). Interestingly, there was no correlation between “total immune” and PFS (Figure 2B), suggesting that patients with longer PFS don't necessarily have a larger total immune infiltrate.

### Relationship of Top Differentially Expressed Genes With Overall Survival

**Figure 3:**
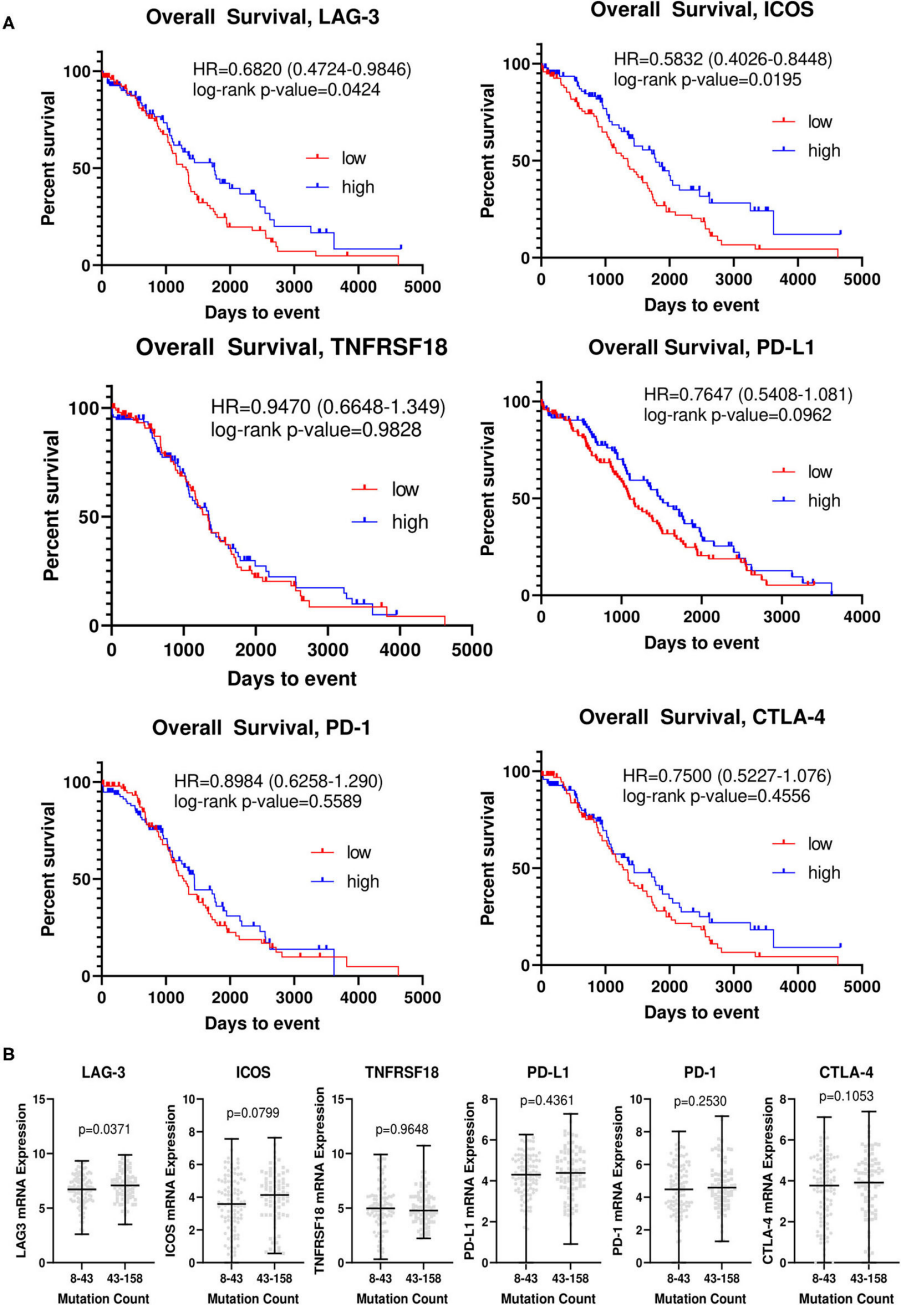
LAG-3 and ICOS are associated with overall survival and mutation counts in The Cancer Genome Atlas ovarian cancer cohort. **(A)** Kaplan-Meier curves for LAG-3, ICOS, CTLA-4, PD-1, PD-L1, and TNFRSF18 were generated using TCGA-OV dataset. The top and bottom quartiles of expression (*n* = 95/group) were used to define “high” vs. “low” expressing groups. Log-rank hazard ratios (HR) and *p*-values are reported, with 95% confidence intervals in parentheses. **(B)** TCGA-OV dataset was separated into low (8–43) and high (43–158) mutation count groups by median mutation count. mRNA expression for each gene (RNA seq V2 RSEM) was determined for each group, with the black bar indicating median mRNA level for each group. *P*-value was determined by two-tailed, unpaired student *t-*test.

We analyzed The Cancer Genome Atlas ovarian cancer cohort (TCGA-OV), and generated Kaplan-Meier curves for overall survival for each of the top differentially expressed genes (Figure 3A). In this larger, somewhat less homogeneous cohort, patients with higher levels of LAG-3 and ICOS had improved OS, with an HR of 0.6820 [0.4724–0.9846] (*p* = 0.0424) for LAG-3 and an HR of 0.5832 [0.4026–0.8448] (*p* = 0.0195) for ICOS. No significant differences were determined for the other candidate genes. Small differences in mean LAG-3 (*p* = 0.0371) and ICOS (*p* = 0.0799) mRNA transcript levels were observed in patients with low vs. high mutation counts, although this difference was not significant for ICOS (Figure 3B).

### Correlation of Top Differentially Expressed Genes Between Each Other and With Immune Cell Subsets

**Figure 4:**
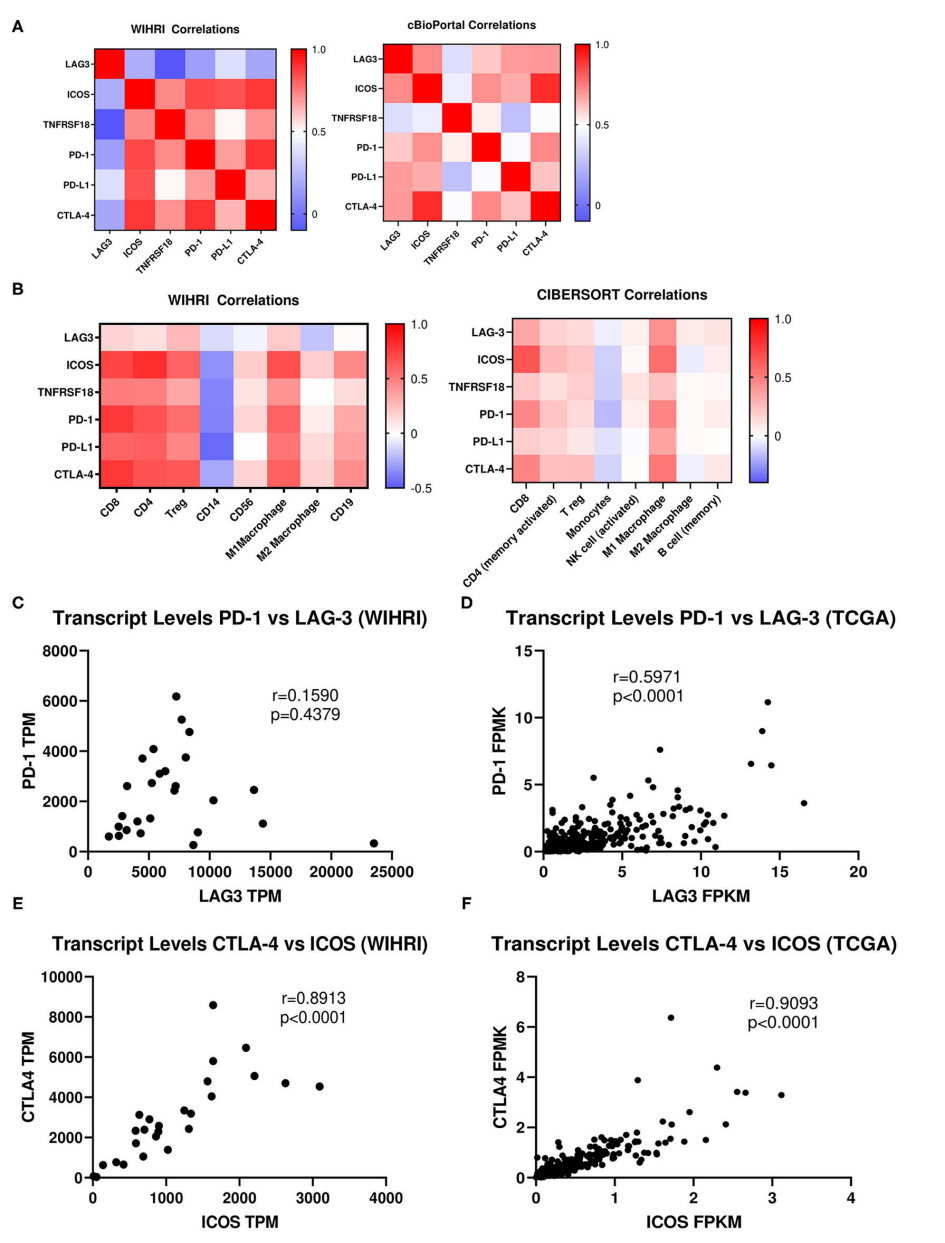
Correlation analysis of immune response genes and individual immune cell subsets. **(A)** Spearman rank correlation analysis was used to determine the relationship between the top differentially expressed genes among each other in all 26 patient samples in our cohort (WIHRI), as well as in TCGA-OV cohort. Heat maps illustrating the strength of the correlations (*r* values) were generated. **(B)** Spearman rank correlation analysis was used to determine the relationship between immune co-receptors and immune cell subsets in the WIHRI cohort (left) and TCGA-OV cohort, using CIBERSORT data (right). **(C,D)** Spearman rank correlation analysis of PD-1 and LAG-3 in the WIHRI and TCGA cohort. **(E,F)** Spearman rank correlation analysis of CTLA-4 and ICOS in the WIHRI cohort and TCGA cohort. Spearman *r* and *p*-values are indicated on each graph.

The top differentially expressed genes were correlated amongst each other and with immune cell subsets. No significant correlations emerged for LAG-3 with the rest of the top differentially expressed immune genes. Conversely, there were strong and significant correlations between ICOS, TNFRSF18, PD-1, PD-L1, and CTLA-4 amongst each other (*r* > 0.5, *p* < 0.001). Likewise, similar correlations between immune genes were observed in TCGA data, except LAG-3 also emerged as significantly correlated, although the strength of LAG-3 correlations was weaker than for ICOS (*p* < 0.0001 for all correlations) (Figures 4A,C–F). Because of reported relationships between PD-1 and LAG-3, as well as between ICOS and CTLA-4, we further examined these relationships in our cohort vs. TCGA. We observed a similar “fan” pattern in the correlation of LAG-3 and PD-1 in our cohort vs. TCGA, although the relationship between these two genes in TCGA dataset emerged as statistically significant (*r* = 0.5971, *p* < 0.0001), likely due to the large number of samples in the TCGA dataset (Figures 4C,D). We also observed a strong correlation between ICOS and CTLA-4 in both our cohort (*r* = 0.8913, *p* < 0.0001) and the TCGA ovarian cancer cohort (*r* = 0.9093, *p* < 0.0001) (Figures 4E,F).

We then performed correlations between the top differentially expressed genes and each immune cell subset in our cohort. There were no significant correlations between LAG-3 and any of the immune cell subtypes, while ICOS, TNFRSF18, PD-1, PD-L1, and CTLA-4 all significantly correlated (*p* < 0.05) to CD8, CD4, and T_regs_, with the exception of TNFRSF18 and T_regs_. There was a trend toward inverse correlation with all genes to CD14+ monocytes, but only PD-L1 significantly inversely correlated (*p* = 0.0230). No immune genes were significantly correlated to CD56+ NK cells or M2 macrophages. ICOS, TNFRSF18, PD-1, PD-L1, and CTLA-4 all significantly positively correlated to M1 macrophages (*p* < 0.05). ICOS and CTLA-4 were both significantly associated with CD19+ B cells (*p* < 0.05). The pattern of correlations between immune genes and cell types was strikingly similar when analyzing TCGA data, using TIMER (CIBERSORT) to generate correlations between immune cell composition and specific immune genes, although the strength of the correlations was overall weaker (Figure 4B).

### PVRL2 Is the Most Highly Expressed Transcript in Ovarian Cancer Tissue and Is Upregulated in Ovarian Cancer Compared to Normal/Benign

**Figure 5:**
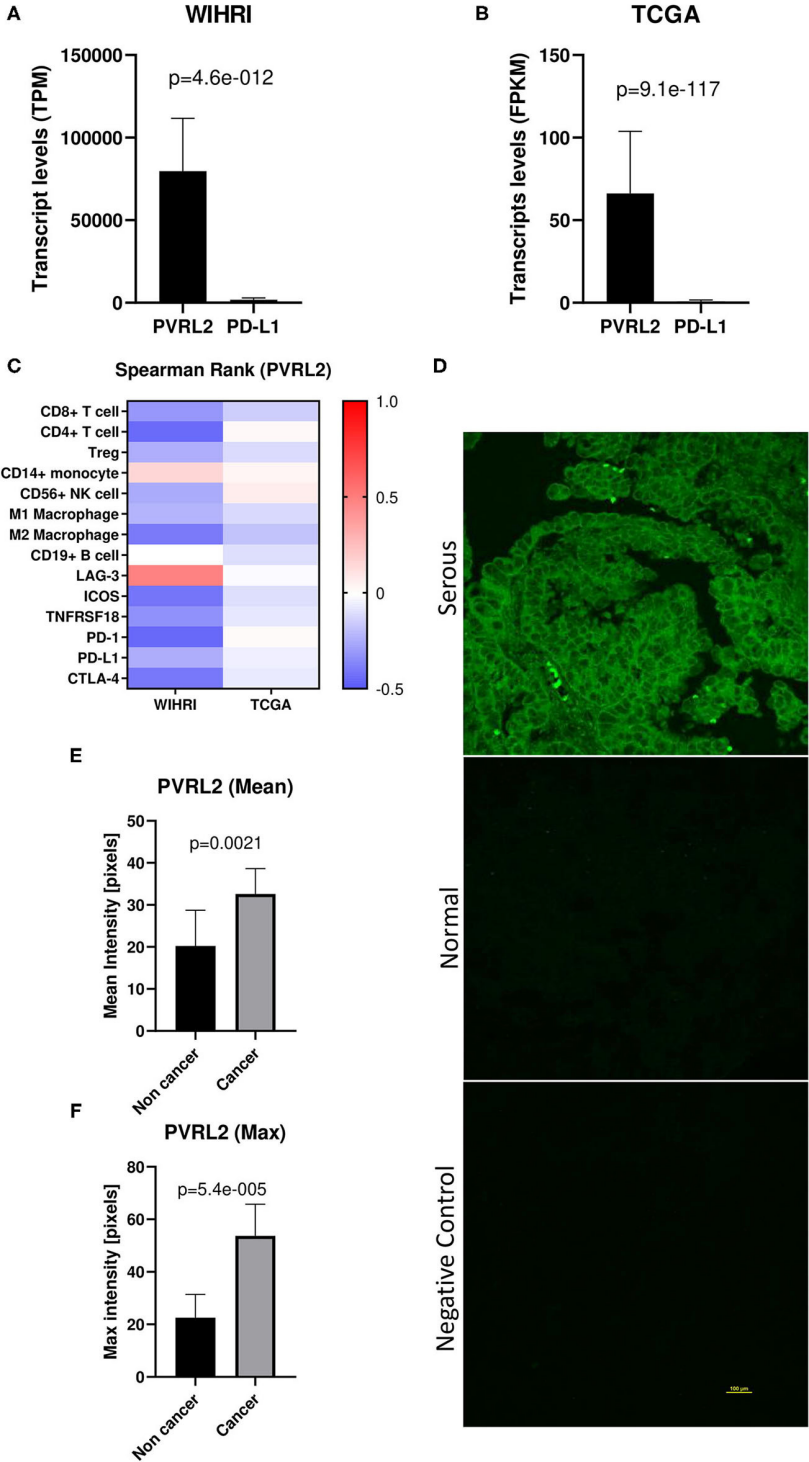
PVRL2 overexpression in high grade serous ovarian cancer. **(A,B)** Transcript levels of PVRL2 vs. PD-L1 in our cohort and TCGA cohort. *p*-values were determined by two-tailed paired student *t*-test. **(C)** Heat map of Spearman rank correlations between PVRL2 and individual immune cell subsets and top differentially expressed immune response genes in WIHRI and TCGA cohorts. Spearman *r* values are indicated by color. **(D)** Representative immunohistochemistry staining of PVRL2 in HGSOC and normal/benign ovaries. Negative control is secondary antibody alone. **(E,F)** Mean and maximum PVRL2 intensity (pixels) in serous ovarian cancer vs. normal/benign tissue.

PVRL2 was found to be the most abundantly expressed transcript out of all immune response genes and ligands within the modeling analysis. Interestingly, when we compared mean expression of PVRL2 to PD-L1, the most commonly studied immune ligand in EOC, we observed over 44-fold higher levels of PVRL2. This was also observed in TCGA-OV dataset, with 82-fold higher levels of PVRL2 compared to PD-L1 (Figures 5A,B). Next, we examined the correlation of PVRL2 to immune cell subsets and found that PVRL2 expression significantly inversely correlated to CD4+ T cells, CD19+ B cells, and M2 macrophages (*p* < 0.05) (Figure 5C). Examining PVRL2's expression with the top regulated immune response genes revealed a significant inverse relationship with PD-1, ICOS and CTLA-4, and a positive correlation with LAG-3 (*p* < 0.05 for all). The trend toward negative correlations between PVRL2 and immune genes and cell types was also observed for TCGA data, although only the correlations of PVRL2 with CD8+ T cells and M2 macrophages were significant (*p* < 0.05). These data sharply contrast with data for PD-L1, which was strongly and significantly correlated with all top immune response genes except LAG-3, as shown in Figure 4A. Next, we performed PVRL2 fluorescent immunohistochemistry on benign/normal (*n* = 5) and HGSOC (*n* = 15) samples. Mean and maximum intensity was 1.6-fold and 2.4-fold greater, respectively, in HGSOC compared to normal/benign tissue (*p* = 0.0021, *p* = <0.00001) (Figures 5D–F), which is in agreement with published studies (29, 30).

## Discussion

This multidimensional immune modeling analysis revealed that a signature of CTLA-4, LAG-3, and T_regs_ was significantly higher in patients with improved clinical outcomes in a subset of patients with HGSOC. While further studies are needed to make any firm conclusion about this multidimensional signature, this study serves as proof-of-principle that immunological signatures can be prognostically informative, and also serves to highlight potential unique immunological responses in ovarian cancer patients.

In addition to PD-1, CTLA-4 is one of the most clinically studied immune checkpoint receptors in EOC, with six open Phase I/II clinical trials evaluating the combination of anti-PD-1/PD-L1 and CTLA-4 therapy (31). While the clinical significance of targeting CTLA-4 has been well-studied, its prognostic value has not previously been investigated in HGSOC. Likewise, LAG-3, whose transcript level we also found to be higher in patients with longer PFS, is a co-inhibitory receptor that has received more attention recently in EOC. Preclinical studies have demonstrated the efficacy of targeting LAG-3 in combination with immune checkpoint receptors in EOC, and Phase I/II development of a LAG-3 monoclonal antibody is currently being investigated in pancreatic, breast, melanoma, and other solid tumors, including ovarian cancer (32). While studies have reported no association between patient outcomes and LAG-3 expression in ascites or residual patient tumors after neoadjuvant chemotherapy (32, 33), the relationship between LAG-3 expression in naïve tumors and clinical outcomes has not been previously studied.

T_regs_ are known to play a particularly important role in ovarian cancer pathogenesis. Intriguingly, it has been reported that response to immunotherapy is exceptionally challenging due to the fact that EOC tumors exhibit elevated levels of highly activated T_regs_ (31), with a distinct phenotype exhibiting higher levels of FOXP3, PD-1, 4-1BB, and ICOS compared to melanoma tumoral T_regs_, while also displaying increased suppressive capabilities against cytotoxic T cell proliferation (34). Despite T_regs_ contributing to challenges with immunotherapy response in EOC, our study identified T_regs_ as part of the multidimensional biomarker for the long PFS group of patients. Contradictory reports exist on the prognostic significance of T_regs_ in ovarian cancer, with one study reporting a positive association between levels of T_regs_ in primary and metastatic lesions and patient outcomes, and another determining a negative association between T_regs_ and survival in all stages of disease (35, 36). While further studies will be required to understand these discrepant results, the outcomes from our study suggest that T_regs_ may be more abundant in a subset of patients with a longer PFS, although further studies in larger cohorts will be needed to fully evaluate this hypothesis. This observation is in agreement with the fact that T_regs_ tend to infiltrate proportionally with effector T cells, which are known to be prognostically favorable in ovarian cancer (8, 14, 15). One major caveat to consider in making conclusions regarding the prognostic ability of T_regs_ and effector T cells in ovarian cancer is their localization. Various reports on the effect of intra-tumoral vs. stromal T cell localization exist, with some reporting that intra-tumoral localization improves prognostication, while others determined that averaging effector T cells through the tumor and stroma improves prognostication (14, 15).

While we are the first to report a combined prognostic significance of CTLA-4, LAG-3, and T_regs_, a major limitation of our study is the small sample size. Furthermore, all patients in our cohort did not receive the exact same therapy, as some patients were treated with bevacizumab in the maintenance setting. While the effect of the bevacizumab treatment should not be significant enough to change the patients' PFS grouping, in future studies this analysis should be repeated in a larger cohort to allow for separate analysis of patients that have received additional regimens apart from platinum-taxane chemotherapy.

Perhaps one of the most interesting findings from this study is the identification of ICOS's association with improved patient survival in EOC. ICOS appears to be a particularly relevant immune gene in ovarian cancer, as ICOS and LAG-3 were the only genes determined from our multidimensional analysis that were validated independently in TCGA cohort. ICOS is a member of the B7 CD28/CTLA-4 costimulatory family of receptors that play a vital role in T cell immunity (37). As an immune checkpoint agonist, ICOS acts as a counterpart to CD28, strongly enhancing T cell proliferation, survival, and differentiation. ICOS is an interesting co-stimulatory receptor, since its function depends on the T cell subset in which it is predominantly expressed. On T_regs_, it may dampen the immune response, while on cytotoxic T cells, it may promote an immune response (38). While ICOS has been studied in other cancers, its role in EOC has yet to be well-elucidated. To date, there have only been two studies that have examined ICOS in the context of EOC, both of which have described its high expression on T_regs_. Conrad et al. (39) reported that the majority of FOXP3+ T_regs_ in the EOC tumor microenvironment expressed ICOS, were responsible for the stimulation of immunosuppressive plasmacytoid dendritic cells, and were associated with disease progression. As stated earlier, Toker et al. (34) also reported that EOC T_regs_ exhibit a distinct phenotype that have higher levels of ICOS compared to melanoma T_regs_, which may explain why melanoma patients have responded well to ICOS agonist interventions as these might primarily act on cytotoxic T cells in melanoma rather than T_regs_. There is a need for larger studies to fully elucidate the expression profile of ICOS in EOC, in order to determine if it can be efficaciously targeted.

It is interesting to note that patient PFS did not correlate with total immune infiltrate and was not statistically associated with any particular immune cell infiltrate. However, since ICOS correlated well with T cell infiltrate, it is still possible that ICOS is prognostically favorable because of preferential increases in T cell infiltrates in patients with improved survival. However, LAG-3 did not correlate well with any immune cell populations in our cohort. These findings suggest that increased immune infiltrate alone is probably not responsible for the observed prognostic ability of these T cell co-receptors. Moreover, other T cell co-receptors examined, such as TIM-3 and BTLA, showed no association with survival, suggesting a unique role for ICOS and LAG-3 in HGSOC.

Finally, results from our immune modeling analysis revealed PVRL2 as the most abundantly expressed immune factor. PVRL2 has been previously shown to be overexpressed in ovarian cancer (29, 30, 40), which we confirmed in a small independent cohort. It is a member of the nectin and nectin-like family that contains receptors such as DNAM-1 (CD226), CD96, T cell immunoreceptor with Ig and ITIM domains (TIGIT), and poliovirus receptor related immunoglobin (PVRIG). PVRL2 binds with a higher affinity to PVRIG rather than TIGIT, which favors the ligand poliovirus receptor (PVR) and only weakly binds to PVRL2 (29). Furthermore, it was found that out of all cancer types studied, ovarian cancer exhibited the highest percentage of PVR-PVRL2+ cells (29), emphasizing a specific overexpression of PVRL2 unique to ovarian cancer.

While PVRL2 was universally highly expressed and no significant relationship existed between PVRL2 levels and PFS, results of the above described studies suggest that anti-PVRIG/PVRL2 based therapy has the potential to greatly impact EOC patients whose tumors lack PD-L1 expression. PD-1/PD-L1 targeting has been well-studied in EOC, despite the fact that only 33% of high grade ovarian cancer patients are considered PD-L1 positive and no significant correlation exists between PD-L1 levels and survival (41). Overall, while the PD-1/PD-L1 axis remains the most frequently investigated clinical immunotherapy target in EOC, it is still uncertain which subset of EOC patients will most benefit from this therapy since response rates remain low and expression analysis studies have shown conflicting data regarding the relationship of PD-1/PD-L1 and clinical outcomes (42). Our data reports an over 44-fold higher expression of PVRL2 compared to PD-L1 in our advanced stage cohort, highlighting the importance of further investigation of this pathway in EOC. Overall, the PVRIG/PVRL2 axis represents a potential therapeutic target that may aide in improving EOC patient response to immunotherapy.

## Conclusions

Our immune modeling analysis revealed that CTLA-4, LAG-3, and T_regs_ are more abundant in HGSOC patients with longer PFS. ICOS was also significantly more highly expressed in patients with longer PFS, and strongly correlated with CTLA-4, PD-1, and specific subsets of immune cell infiltration. High ICOS and LAG-3 levels were significantly associated with longer survival in the ovarian cancer TCGA cohort. PVRL2 was the most abundantly expressed transcript in the ImmunoPrism analysis, and was overexpressed in a cohort of HGSOC tissue compared to benign/normal samples. Our findings highlight the idea that immunological signatures are related to patient outcomes in HGSOC.

## Data Availability Statement

The datasets presented in this study can be found in online repositories. The names of the repository/repositories and accession number(s) can be found in the article/Supplementary Material.

## Ethics Statement

The studies involving human participants were reviewed and approved by Women & Infants Hospital Institutional Review Board. A waiver of written informed consent was obtained for this study in accordance with the national legislation and the institutional requirements.

## Author Contributions

JR, JO, and NJ designed the study. KM, MW, and NJ executed experiments. KM and EL performed chart reviews. JR and NJ performed statistical analyses. JO obtained all samples and assured they met minimum quality standards. NL oversaw the ImmunoPrism assay and analysis and provided figures, tables, and methods pertaining to ImmunoPrism results. JR oversaw all aspects of the study. JR, NJ, and KM prepared the manuscript, which was edited by NL. All authors reviewed and approved the final version.

## Conflict of Interest

NL is an employee of Cofactor Genomics. The remaining authors declare that the research was conducted in the absence of any commercial or financial relationships that could be construed as a potential conflict of interest.
