# Supplementary material for: Immune Modeling Analysis Reveals Immunologic Signatures Associated With Improved Outcomes in High Grade Serous Ovarian Cancer
Source: Front Oncol. 2021 Mar 5;11:622182. doi: 10.3389/fonc.2021.622182 (PMC7973276; doi:10.3389/fonc.2021.622182)
Supplement: Supplementary file 1 [file Table_1.docx]

**Supplementary Table 1. Outliers in the ImmunoPrism Analysis.** The “Multidimensional Value” refers to where the patient falls on the Multidimensional Biomarker Assessment graph in Figure 1.

| Group | Stage | Grade | Age Range | Received Maint. Bev | PFS  (with bev | OS  (with bev) | HE4 | CA125 | Debulking | Multi-dimensional Value |
| --- | --- | --- | --- | --- | --- | --- | --- | --- | --- | --- |
| Long | IIIC | 3 | 70-79 | Yes | 62 (51) | 77 (66)+ | N/A | 0-499 | Optimal | 0.67 |
| Long | IIIC | 3 | 50-59 | Yes | 76 (64)+ | 76 (64)+ | 0-499 | 500-999 | Optimal | 0.67 |
